# A systematic review and meta-analysis of the association between alexithymia and suicide ideation and behaviour

**DOI:** 10.1016/j.jad.2019.05.013

**Published:** 2019-07-01

**Authors:** Laura Hemming, Peter Taylor, Gillian Haddock, Jennifer Shaw, Daniel Pratt

**Affiliations:** a Division of Psychology and Mental Health, School of Health Sciences, University of Manchester, UK; b Manchester Academic Health Sciences Centre, Manchester, UK; c Greater Manchester Mental Health NHS Foundation Trust, Manchester, UK

**Keywords:** Suicide, Alexithymia, Depression, Meta-analysis

## Abstract

**Background:**

Alexithymia reflects a difficulty with identifying and expressing emotions. This experience has been proposed as having an association with suicide ideation and behaviour. This review aimed to synthesise the evidence to establish the bivariate and multivariate relationships between alexithymia, and its subcomponents, with suicide ideation and behaviour.

**Methods:**

Search terms related to alexithymia and suicide ideation and behaviour were searched across nine prominent databases in May 2018. Studies were eligible for inclusion if they reported original empirical quantitative findings from adult samples, used a validated measure of alexithymia, and any measure of suicide ideation or behaviour.

**Results:**

Thirty-four studies were eligible for inclusion in this review. The review found a large effect size for the relationship between alexithymia and suicide ideation (*r* = 0.54, 95% CI= 0.40–0.65) and a small effect size for the relationship between alexithymia and suicide behaviour (*r* = 0.25, 95% CI = 0.16–0.34).

**Limitations:**

A high level of heterogeneity was found in the meta-analysis meaning that results should be interpreted with caution.

**Conclusion:**

A positive association was found between alexithymia and suicide ideation and, to a lesser extent, behaviour across a range of clinical and general population samples. This review has potentially important clinical implications, and promotes the need for suicide prevention to focus on emotion regulation skills.

## Highlights

• Alexithymia is more closely related to suicide ideation than to suicide behaviour.
• The subcomponents of difficulty identifying and describing feelings are more closely related to suicide ideation and behaviour than the subcomponent of externally oriented thinking.
• The relationship between alexithymia and suicide ideation appears robust when controlling for confounding variables, though the relationship between suicide behaviour and alexithymia is not so robust.
• Future research should aim to examine causality in this relationship through using longitudinal designs.

## 1 Introduction

Globally, approximately 800,000 people die by suicide every year (WHO, 2014). Suicide is the most common cause of death for men aged 20–49 years in England and Wales (Office for National Statistics, 2016) and it is estimated that one person in fifteen has made a suicide attempt at some point in their life (McManus et al., 2016). Further to this, lifetime prevalence of suicide ideation in Europe is estimated at 5.6% (Hardt et al., 2015). Suicide ideation has been found to be related to emotional distress (Vilhjálmsson et al., 1998), and more severe psychopathological symptoms (Reinherz et al., 2006). It has therefore been advocated that suicide ideation be considered as a key clinical outcome in its own right (Tarrier et al., 2008).

Suicidality is operationalised in this review as comprising three main factors; suicide ideation, suicide behaviour and suicide risk. In this review, suicide ideation refers to thoughts, plans and wishes to end life through suicide either alongside or separate from attempting suicide (Beck et al., 1972). Suicide behaviour encompasses a wide range of self-inflicted behaviours which could potentially end a person's life. This may include but is not limited to; self-harm behaviours, suicide attempts, completed suicides and expressing suicidal plans (Silverman et al., 2007). In this review, suicide behaviour specifically refers to suicide attempts only; other aspects of suicide behaviour mentioned above are unfortunately beyond the scope of this review. Finally, in this review, suicide risk reflects a composite measure of various risk factors previously found to be associated with those who have attempted suicide compared to those who have not (Plutchik et al., 1989).

Research investigating causes of suicide should consider factors that predict suicide ideation, and those that may explain the transition from suicide ideation to suicide behaviour (Klonsky and May 2015). This is supported by findings that the correlates of suicide ideation and behaviour could be markedly different. For instance, a recent meta-analysis suggested that whilst factors such as depression and hopelessness distinguished people experiencing suicide ideation from people who had never experienced suicidality, these same factors did not distinguish between those experiencing suicide ideation and those who acted on these thoughts (May and Klonsky, 2016). It is therefore important that research exploring the clinical correlates of suicide aims to look specifically at correlates of suicide ideation and suicide behaviour separately.

It has been frequently observed that a common antecedent to suicidal thoughts and behaviours is the experience of unmanageable emotional distress (Dour et al., 2011, Shneidman, 1998). Furthermore, individuals who experience greater difficulty in regulating high levels of emotional distress are more likely to die by suicide (Pisani et al., 2013). It has therefore been suggested that emotional dysregulation may have a considerable impact on suicidal thoughts and behaviours. Emotion dysregulation has been defined as a multidimensional construct involving maladaptive ways of responding to emotions, regardless of their intensity (Gratz and Tull, 2010). Evidence has now been found for a relationship between suicidal thoughts and behaviours and emotional under-regulation strategies, such as rumination (Morrison and O'connor, 2008), as well as emotional over-regulation strategies, such as emotional avoidance (Najmi et al., 2007).

Related to emotion dysregulation is the concept of alexithymia, defined as the inability to identify or express emotions (Sifneos, 1973). Whilst originally perceived to be a personality trait, it has since been defined as a form of emotion regulation (Salminen et al., 2006, Taylor, 2000). Since its inception in the field of psychosomatics, alexithymia has been described as having five main components: i) a difficulty in identifying one's emotions ii) a difficulty in describing self-feelings verbally iii) a reduction or incapability to experience emotions iv) an externally orientated cognitive style and v) poor capacity for fantasising or symbolic thought (Taylor and Bagby, 2000). Alexithymia is most commonly measured using the Toronto Alexithymia Scale (Bagby et al., 1994), which comprises three main factors. Difficulty identifying feelings (DIF) assesses a person's ability to recognise their emotions, difficulty describing feelings (DDF) assesses a person's ability to communicate their emotions to others, and externally oriented thinking (EOT) assesses a person's tendency to focus their attention externally.

One concept that may link alexithymia to suicide ideation and behaviour is depression. Indeed, depressed patients have been found to experience a greater severity of alexithymia than individuals with other psychiatric disorders (Bankier et al., 2001, Leweke et al., 2012). Several studies have found a relationship between alexithymia and depression with a recent meta-analysis finding that alexithymia severity scores were moderately correlated to scores of depression severity in a range of clinical and general population samples (*r* = 0.46; Li et al., 2015). Given the close relationship between depression and suicide behaviour (Blackmore et al., 2008), it therefore seems plausible that depression might play a role in the posited relationship between alexithymia and suicidal thoughts and behaviours. Indeed it has been found that depression mediates the relationship between alexithymia and suicide risk (DeBerardis et al., 2017).

In addition to alexithymia impacting on suicidal thoughts and behaviours via depression, this relationship has also been found to exist independently of depression (e.g. Kim et al., 2016). It has been suggested that individuals with alexithymia may experience emotional information as overwhelming and confusing, which can lead to feelings of helplessness (Foran and O'Leary, 2013). Furthermore, effective emotion regulation is centred on both the ability to identify one's feelings (emotional clarity) alongside the ability to respond and recover from negative emotions (access to effective strategies; (Gratz and Roemer, 2004)). Thus, for individuals with poor emotional clarity, it is more difficult to progress to effectively regulate these emotions. Conversely, individuals experiencing alexithymia frequently utilise maladaptive coping strategies, such as social isolation, behavioural disengagement and emotional inhibition (Velasco et al., 2001). It is therefore hypothesised that individuals experiencing alexithymia may become motivated towards suicide ideation and behaviour due to an impaired / limited awareness of alternate ways of coping with the unnameable feelings they are experiencing.

### 1.1 Aims of the study

A range of studies have now linked alexithymia with suicide ideation and behaviour, however, there has been no synthesis of the research to date which examines this relationship. There are therefore several unanswered questions about this relationship, including whether particular features of alexithymia are specifically related to suicide ideation and behaviour, and whether alexithymia is more closely related to the development of suicide ideation or behaviour. Further to this, it is unclear whether specific mechanisms play a role in the facilitation of suicidal thoughts and behaviours in those who are experiencing alexithymia. The current systematic review therefore aims to synthesise the evidence on the relationship between alexithymia and suicide ideation and/or behaviour and to compare the relationships between alexithymia and suicide ideation / behaviour. Specifically, it was hypothesised that:

1 There will be a relationship between alexithymia, and its subcomponents, with suicidal ideation in adults
2 There will be a relationship between alexithymia, and its subcomponents, with suicidal behaviour in adults

## 2 Materials and methods

A protocol for the current systematic review can be found on the CRD Prospero website (ref: CRD42017069076)

### 2.1 Search strategy

The review was conducted and reported as per the Preferred Reporting Items of Systematic Reviews and Meta-Analyses (PRISMA) guidelines (Moher et al., 2009). The following databases were searched for eligible studies: PsycINFO, EMBASE, MEDLINE, PubMed, Web of Science ASSIA, Sociological Abstracts, CINAHL, ComDisDome and Cochrane Trials Library.

The first search term related to alexithymia and was “alexithymi*”. This search term was specific to the concept of alexithymia, due to the review focussing only on the relationship between alexithymia and suicide ideation and behaviour, as opposed to similar but broader constructs such as emotion dysregulation.

The alexithymia search term was linked with the Boolean operator ‘AND’ to a set of search terms that related to suicide ideation and behaviour: “suicid*” OR “parasuicid*” OR “suicid* ideation” OR hopeless* OR “self-harm*” OR “deliberate self-harm*” OR “DSH” OR “attempt* suicide” OR “overdos*” OR “self-injur*” OR “self-destruct*” OR “self-inflict*” OR “self-mutilat*” OR “self-poison*” OR “self-immolat*” OR automutilat* OR “auto-mutilat*” OR “self-cut*” OR “auto-destruct*” OR “non-suicidal self injur*” OR “NSSI”. Given the difficulties in differentiating between self-harm and suicidal behaviour, the suicide search terms were purposefully broad to include papers that may report both on suicide behaviour and self-harm. These search terms were used in initial screening searches, and were found to elicit all eligible studies.

Full database searches were conducted in May 2018, with no limit on the year of published studies to be included in the review. To ensure the search was as inclusive as possible, including the grey literature, ‘citation chaining’ was performed, whereby the ‘cited-by’ function in Google Scholar was used to identify any eligible studies that had cited studies included in the review, and the reference lists of included studies were screened to identify any studies that the search terms had not identified. Despite critiques that Google Scholar may contain inadequacies (Vine, 2006), Google Scholar has been found to be of sound utility in its search functions, in fact often returning unique citations (Falagas et al., 2008).

### 2.2 Eligibility criteria

The following criteria were used to identify eligible studies:

1 An empirical study in English language
2 Included a validated measure of alexithymia (validated measures had to have at least one other study published on the psychometric properties of the scale)
3 Included any measure of suicide ideation or behaviour
4 Reported on the relationship between alexithymia and suicide ideation or behaviour
5 Studies in which at least two thirds of the sample were aged sixteen or over (deciphered either from the age range, or where this information was not available, from calculating the mean age minus one standard deviation)

Samples aged sixteen years or under were excluded in the present review on the theoretical basis that emotions and concurrent emotion regulation strategies are not fully formed at this young age (Zeman et al., 2006), and thus alexithymia may not be an appropriate concept for this age group. Further to this, there is an empirical issue in the measurement of alexithymia in this age group, as it has been noted that the Toronto Alexithymia Scale has not yet been validated with this age group and in fact demonstrated poor internal consistency with younger age groups (Parker et al., 2010).

Studies were excluded on the following basis:

1 Did not report original findings (e.g. reviews, book chapters, clinical guidelines, conference abstracts, letters, case studies, study protocols)
2 Use of qualitative methods only

### 2.3 Study selection and risk of bias

The first author screened the search results at title and abstract level against the identified eligibility criteria. In addition, an independent researcher screened 20% to provide a measure of the reliability of the screening process. The two researchers had an agreement rate of 97%, (Cohen's Kappa = 0.92, *p*<.001). The first author then screened eligible results at full study level, with an independent researcher also screening 20%. The two researchers had an agreement rate of 95% (Cohen's Kappa = 0.87, *p*<.001). Any disagreements that could not be resolved through consensus were discussed with a third reviewer (DP).

The first author then extracted data using a pre-piloted data extraction form. The data extraction form was piloted with three eligible studies. The data extraction form elicited the following information from each study: title, authors, year of publication, country, funding sources, conflicts of interest, research aims, research design, measures used, analyses conducted, number of participants, participant response rate, sample characteristics and demographics, results of analysis.

A revised version of the AXIS tool (Downes et al., 2016) was utilised to assess risk of bias for all cross-sectional studies included in the review. An additional question was added for case-control studies (“Are cases clearly defined and differentiated from controls?”). In addition, an independent researcher assessed risk of bias in twenty percent of full studies to provide a measure of reliability of this assessment process and there was good agreement between the two raters.

### 2.4 Synthesis of results

Meta-analyses of standardised mean differences and correlations were performed using the MetaXL v5.3 (Barendregt and Doi, 2010) add-in for Microsoft Office Excel. The effect size used in the meta-analyses was the correlation coefficient. The Fisher's Z transformation was applied to correlation coefficients to stabilize their variance prior to inclusion within the meta-analysis (Borenstein et al., 2011). The results were then converted back into correlation coefficients to aid interpretation. The standard error for these effect sizes was calculated from the sample size (see equations in Borenstein et al., 2011). Where correlation coefficients were not available, Cohen's D scores were calculated to ascertain the effect size of mean differences. The Cohen's D score was then converted into a correlation coefficient.

Meta-analyses were performed using a random-effects model due to expected differences between studies in the definition and measurement of suicide ideation and behaviour. The I^2^ statistic was calculated to estimate inconsistency, as this statistic estimates the proportion of variance explained by heterogeneity (Higgins and Thompson, 2002). According to Higgins and Thompson (2002), an I^2^ value of over 75% indicates high heterogeneity. Heterogeneity was addressed by examining possible outliers via visual inspection of forest plots, and where appropriate, using post-hoc sensitivity analysis to remove these outliers.

Effect sizes of the relationship between alexithymia and suicide ideation / behaviour were compared by contrasting effect sizes and their precision using Cohen's (Cohen, 1988) framework of effect sizes as a guideline. Formal decision rules were not deemed appropriate due to the differences in effect sizes needing to be considered within the wider context of study characteristics and methodological quality.

## 3 Results

Authors of papers which reported on the relationship between alexithymia and suicide ideation or behaviour but did not report a statistical analysis were contacted to provide the statistical analysis of this relationship. A total of fourteen authors were contacted. Ten authors did not reply, three replied explaining they were unable to provide the analysis and one replied supplying the analysis requested, rendering this paper eligible for inclusion (Meaney et al., 2016).

**Fig. 1:**
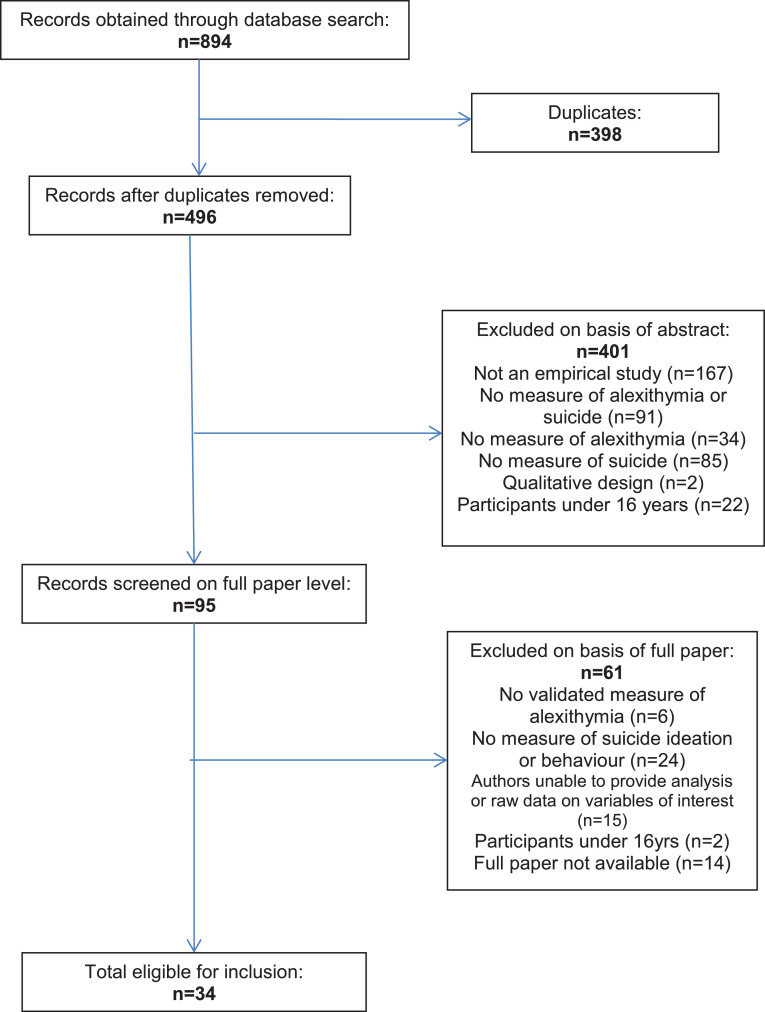
PRISMA flow diagram of systematic search.

A flowchart of each stage of the database search was produced, with the search returning a total of 894 studies (Fig. 1).

### 3.1 Study characteristics

A total of 34 studies were included in the review which incorporated data from 10,104 participants. Table 1 summarises the characteristics of included studies in the review. Eleven studies assessed the relationship between alexithymia and suicide ideation, twelve the relationship between alexithymia and suicide behaviour, eight the relationship between alexithymia and both suicide ideation and behaviour and three the relationship between alexithymia and suicide risk.

**Table 1:** Characteristics of included studies.

Authors and year	Country	Research design	N	Sample characteristics	Age (mean, SD)	Gender - male N (%)	Alexithymia measure	Suicide ideation measure	Suicide behaviour measure	Suicide risk measure
Carano et al. (2012)	Italy	Cross-sectional	80	Binge eating disorder outpatients	39.5 (8.2)	38 (48%)	TAS-20	SSI	History taking – lifetime attempt	
Chang et al. (2016)	Taiwan	Case-control	1077	Suicide attempt / threat patients, controls	39.0 (16.4)	446 (41%)	TAS-20	BSRS	Clinical records – lifetime attempt	
De Berardis, Campanella, et al. (2013)	Italy	Cross-sectional	72	Panic disorder outpatients	25.3 (3.3)	37 (51%)	TAS-20	SSI		
De Berardis, Conti, et al. (2013)	Italy	Cross-sectional	30	Non-affective psychosis patients	25.9 (6.0)	13 (43%)	TAS-20	SSI		
De Berardis et al. (2008)	Italy	Cross-sectional	145	Major depression outpatients	28,5 (5.7)	69 (48%)	TAS-20	SSI		
De Berardis et al. (2017)	Italy	Cross-sectional	70	GAD outpatients	28.2 (5.3)	34 (49%)	TAS-20	SSI		
De Berardis et al. (2015)	Italy	Cross-sectional	104	OCD outpatients	32.1 (8.0)	52 (50%)	TAS-20	SSI		
De Berardis et al. (2014)	Italy	Case-control	79	OCD outpatients, controls	28.7 (8.0)	43 (54%)	TAS-20	SSI		
Evren and Evren (2006)	Turkey	Cross-sectional	154	Substance dependent patients	31.9 (11.7)	154 (100%)	TAS-20		CANQ – lifetime attempt	
Evren et al. (2009)	Turkey	Cross-sectional	159	Substance dependent patients	36.9 (8.4)	159 (100%)	TAS-20		CANQ – lifetime attempt	
Ghorbani et al. (2017)	Iran	Case-control	305	Alcoholic outpatients, controls	33.4 (10.3)	214 (70%)	TAS-20	SSI	History taking – lifetime attempt	
Gulec et al. (2014)	Turkey	Case-control	144	Conversion disorder patients, controls	Not reported (age range 18–64)	29 (20%)	TAS-20		Clinical records – lifetime attempt	
Hamilton (2003)	USA	Cross-sectional	103	Undergraduate students	20.6	22 (21%)	TAS-26			SPS
Haviland et al. (1988)	USA	Cross-sectional	125	Substance dependent in-patients	40.8 (11.2)	125 (100%)	TAS-26		History taking – lifetime attempt	
Hintikka et al. (2004)	Finland	Longitudinal	1563	General population	45.1 (10.5)	649 (42%)	TAS-20	BDI item 9		
Huang et al. (2016)	Taiwan	Case-control	455	Maladjusted soldiers, controls	21.9 (4.5)	429 (95%)	TAS-20	BSRS		
Iancu et al. (2001)	Israel	Case-control	66	Panic disorder outpatients, controls	44.3 (11.7)	26 (40%)	TAS-26			SRS
Iancu et al. (1999)	Israel	Case-control	60	Major depression patients, controls	35 (15.7)	30 (50%)	TAS-26		Clinical records – hospitalised for suicide attempt	
Izci et al. (2015)	Turkey	Case-control	149	Depression patients, controls	41.4 (12.6)	59 (59%)	TAS-20	SfSI	History taking - lifetime attempt	
Kampfer et al. (2016)	Germany	Cross-sectional	155	Somatoform disorder patients	42.2 (15.7)	66 (43%)	TAS-20		History taking – lifetime attempt	
Kim et al. (2016)	Korea	Cross-sectional	81	OCD patients	28.9 (7.6)	50 (62%)	TAS-20	SSI	History taking – lifetime attempt	
Kusevic et al. (2015)	Croatia	Cross-sectional	127	War veterans with PTSD	48.1 (6.6)	127 (100%)	TAS-20		History taking – lifetime attempt	
Laget et al. (2006)	Switzerland	Case-control	570	Suicide attempt patients, controls	27 (8.5)	225 (40%)	TAS-20		Hospitalised suicide attempt patients	
Lester (1991)	USA	Cross-sectional	127	Undergraduate students	21.3 (3.9)	54 (43%)	TAS-26	BDI item 9	History taking – lifetime attempt	
Levi et al. (2008)	Israel	Case-control	173	Suicide attempt patients, controls	37.5 (14.3)	86 (50%)	TAS-20		Hospitalised suicide attempt patients	
Links et al. (2012)	Canada	Longitudinal	120	Psychiatric ward discharged patients	37.5 (11.1)	63 (53%)	TAS-20	SSI	PHI – attempts in the past year	
Loas et al. (2016)	France	Cross-sectional	122	Inpatients with mood or anxiety disorder	44.6 (13.5)	33 (27%)	TAS-20	BDI item 9		
Loftis (2014)	USA	Cross-sectional	550	Undergraduate students	20.5 (3.6)	272 (50%)	TAS-20			SBQ-R
Meaney et al. (2016)	Australia	Cross-sectional	2261	Undergraduate students	24.8 (8.1)	619 (27%)	TAS-20		BSL-supplement – attempts in past week	
Na et al. (2013)	Korea	Case-control	276	Adjustment disorder soldiers, controls	20.9 (1.4)	276 (100%)	TAS-20		Clinical records – attempts since joining armed forces	
Rogers (2015)	USA	Cross-sectional	99	Psychiatric inpatients	40.6 (12.0)	0 (0%)	TAS-20		SADS – attempts in past 6 months	
Sakuraba et al. (2005)	Japan	Case-control	164	Alcoholic patients, controls	53.9 (7.7)	164 (100%)	TAS-26	SSI		
Sayar et al. (2003)	Turkey	Case-control	160	Suicide attempt patients, controls	25.0 (8.3)	26 (23%)	TAS-26	ZSDS item 19	History taking – lifetime attempt	
Wood et al. (2010)	UK	Case-control	179	Traumatic brain injury patients, controls	28.2 (15.0)	118 (66%)	TAS-20	BDI item 9		

BDI = Beck Depression Inventory, BSL-supplement = Borderline Symptom List-supplement: Items for assessing behaviour, BSRS = Brief Symptom Rating Scale, CANQ = Childhood Abuse and Neglect Questionnaire, ISS = Intent Score Scale, LRS = Lethality Rating Scale, PHI = Parasuicide History Inventory, SADS = Schedule of Affective Disorders for Schizophrenia Suicide Module, SBQ-*R* = Suicide Behaviours Questionnaire-Revised, SfSI = Scale for Suicide Ideation, SIS = Suicide Intent Scale, SLS = Suicide Lethality Scale, SPS = Suicide Probability Scale, SRS = Suicide Risk Scale, SSI = Scale of Suicidal Ideation, TAS = Toronto Alexithymia Scale, ZSDS = Zung Self-rating Depression Scale.

Of the 34 studies, 31 were published studies in peer-review journals and three were dissertation theses. Included studies utilised a range of designs; eighteen were cross-sectional studies, fourteen case control and two longitudinal. Included studies took place in a variety of countries worldwide; seven in Italy, five in Turkey, five in USA, three in Israel, two in Taiwan and Korea and one in Finland, Iran, Germany, Croatia, Switzerland, Canada, France, Australia, Japan and the UK. The majority of included studies (70%) were conducted within the past 10 years. Most included studies (85%) sampled clinical populations, with just 5 of the included studies sampling students or general population.

### 3.2 Measures used

All studies used a version of the Toronto Alexithymia Scale (TAS) to assess alexithymia. Four studies used the original TAS-26 scale (Taylor et al., 1985) and the more recent TAS-20 scale (Bagby et al., 1994) was used in the remaining studies.

Included studies used a variety of measures of suicide ideation. The majority of studies (*k* = 11) used the Scale for Suicide Ideation (Beck et al., 1979). Five studies used suicide ideation items from scales of depression including Beck Depression Inventory (Beck et al., 1996) and Zung Self-Report Depression Scale (Zung, 1965). The remaining studies utilised the brief symptom rating scale (*k* = 2) (Lee et al., 2003) or the Scale for Suicide Ideation (*k* = 1) (Linehan and Nielsen, 1981). Each of these scales assesses recent suicide ideation, asking for responses ranging from today up to the past two weeks.

Similarly, a variety of measures were utilised to report suicide behaviour. The majority of studies (*k* = 16) used a bespoke self-report question to measure number of suicide behaviours. An example of such a question is: “In your lifetime, did you ever make a suicide attempt?” (Carano et al., 2012). The time range that suicide behaviours were measured across varied, though most studies asked about lifetime suicide attempts (see Table 1).

Finally, three studies took an aggregate measure of suicide ideation and suicide behaviour, which it was not possible to separate for analysis purposes. These studies are therefore included in the review, in the exploration of the association between alexithymia and suicide ‘risk’. Hamilton (2003) used the Suicide Probability Scale (Cull and Gill, 1989), Iancu et al. (2001) used the Suicide Risk Scale (Plutchik et al., 1989) and Loftis (2014) used the Suicide Behaviours Questionnaire Revised (Osman et al., 2001).

### 3.3 Risk of bias and heterogeneity

The results of the quality assessment using the adapted AXIS tool showed that all studies were of good quality. Despite this, there were some common areas for which it was difficult to assess for risk of bias, due to studies not routinely reporting information. For instance, only two included studies gave information about non-responders, meaning this data bias could not be accounted for in the present review and may therefore affect the validity of the results. Furthermore, none of the included studies gave a justification for the sample size. This, again, may affect the validity of the results in this review, given that ten studies included a sample size of fewer than 100 participants. This may mean that studies in this review were underpowered, which can often lead to Type 2 errors. Out of the 34 included studies in this review, only eight studies explicitly addressed and categorised non-responders. This may mean that the studies included in this review are inherently biased in their samples. However, the majority of studies included in the review did use a selection process which was likely to select participant's representative of the target population, so this may go some way to alleviating this risk of bias.

All 34 studies identified clear aims and objectives, used study designs appropriate for the aims, clearly identified the target populations, measured risk factors and outcomes appropriate to the aims of the study, using instruments that have been trialled, piloted or published previously, discussed limitations of their study, and gave justified conclusions by the results.

According to Higgins and Thompson's (2002) cut-off points, heterogeneity levels were high in the majority of analyses completed (Table 2), supporting the decision to use a random-effects model. It is important to note that due to high levels of inconsistency, the 95% confidence intervals are likely to be more informative than relying only upon point-estimates of effect sizes.

**Table 2:** Heterogeneity of meta-analyses.

Analysis	Number of studies	I^2^ value
Suicide ideation and alexithymia	15	95%
Suicide behaviour and alexithymia	16	85%
Suicide ideation and difficulty identifying feelings	6	79%
Suicide ideation and difficulty describing feelings	5	90%
Suicide ideation and externally oriented thinking	5	89%
Suicide behaviour and difficulty identifying feelings	8	65%
Suicide behaviour and difficulty describing feelings	8	58%
Suicide behaviour and externally oriented thinking	8	63%

**Fig. 2:**
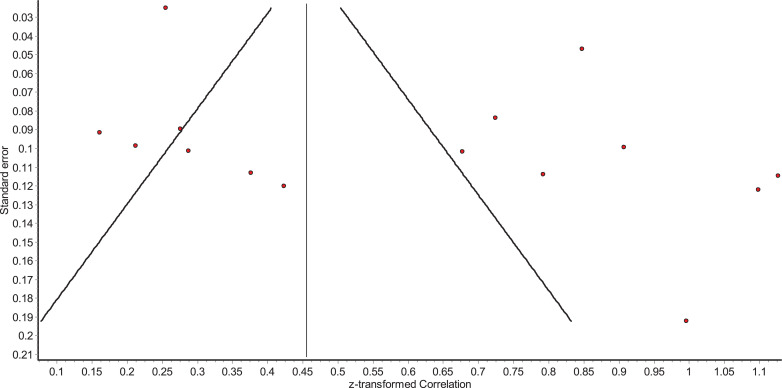
Funnel plot of relationship between alexithymia and suicide ideation.

**Fig. 3:**
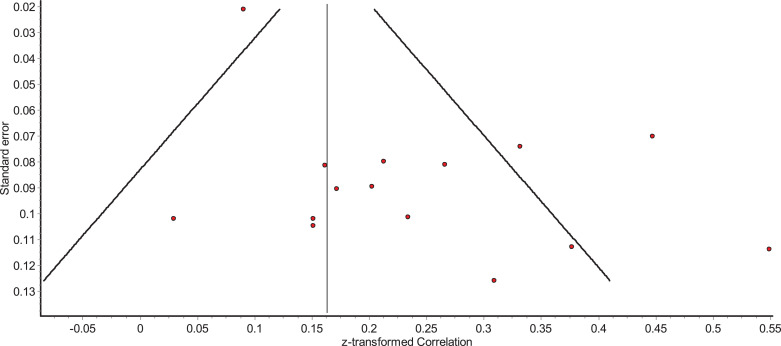
Funnel plot of relationship between alexithymia and suicide behaviour.

For those analyses where more than ten studies were included in the analysis, funnel plots were inspected to identify the possibility of publication bias. All funnel plots appeared asymmetric, though this may have occurred due to the high levels of heterogeneity, potentially caused by differences in study populations (Sterne et al., 2011). An Egger's test of publication bias supported the notion that there was no publication bias in studies that investigated the relationship between alexithymia and suicide ideation (β=3.76, SE=1.87, *p*=.065). For studies that investigated the relationship between alexithymia and suicide behaviour, Egger's test revealed a possibility of publication bias (β=2.15, SE=0.78, *p*=.016). However, it is important to note that there are only fifteen studies included in each Egger's test which can result in individual studies having an inflated effect on the test. The reader is therefore referred to the funnel plots (Figs. 2 and 3).

### 3.4 The relationship between suicide ideation and alexithymia

#### 3.4.1 Suicide ideation and alexithymia: bivariate relationship

**Fig. 4:**
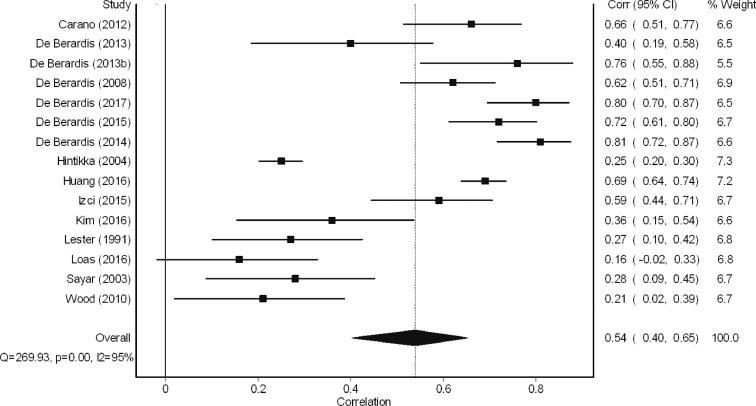
Meta-analysis of relationship between alexithymia and suicide ideation.

A total of 16 studies examined the bivariate relationship between alexithymia and suicide ideation. The meta-analysis revealed a ‘large’ effect size (Cohen, 1988) in the relationship between alexithymia and suicide ideation (*r* = 0.54, 95% CI = 0.40–0.65, Fig. 4). When considering study design, it was found that effect sizes were marginally larger for studies using a between-groups case-control design (difference in alexithymia scores; Cohen's *D* = 1.30, 95% CI =0.50–2.10, *k* = 4. Difference in suicide ideation scores; Cohen's *D* = 1.95, 95% CI = 1.44–2.46, *k* = 6) than for studies using a within-group cross-sectional design correlation design studies (*r* = 0.34, 95% CI =0.18–0.48, *k* = 5). This is likely due to the fact that between-groups design studies often maximise the difference through comparing a group with particularly high scores on a particular trait to a group with particularly low scores on a particular trait. The difference in these groups is therefore often more pronounced than searching for a correlation between variables within one sample. The 95% confidence intervals were much larger for the effect sizes computed for between-group design studies than for within-group design studies, suggesting the within-group studies had a wider range in findings than those which utilised correlation techniques.

As can be seen in Fig. 4, of the nine studies that yielded a ‘large’ effect size, six of these studies were conducted by the same research team in Italy (De Berardis and colleagues). Post-hoc sensitivity analysis was conducted to investigate further the potential effect this group of results may have had upon this meta-analysis. When the findings of all studies conducted by DeBerardis et al. were removed, the effect size of the relationship between alexithymia and suicide ideation was smaller, although still moderate (*r* = 0.41, 95% CI = 0.25–0.55). The inconsistency I^2^ values of this analysis remained the same (I^2^ = 95%).This group of six studies appear no different to the other studies included in the meta-analysis in relation to their risk of bias as reported using the AXIS quality assessment tool (Downes et al., 2016).

It is notable that those studies which used a validated measure of suicide ideation yielded larger effect sizes than the five studies which opted for specific items from validated scales of depression symptoms (Hintikka et al., 2004, Lester, 1991, Loas et al., 2016, Sayar et al., 2003, Wood et al., 2010).

In addition to the data included in the meta-analysis, three studies (Carano et al., 2012, De Berardis et al., 2015, Hintikka et al., 2004) also reported on the mean difference in prevalence of suicide ideation between those who were experiencing alexithymia and those who were not. The odds ratios ranged from 4.59 (95% CI=3.11–6.78) to 94.67 (95% CI=21.96–408.09), indicating a 4 to 95-fold increase in risk of suicide ideation for those experiencing alexithymia. These figures are likely inflated as a result of issues of data separation, and so the more extreme values are unlikely to be robust. Nonetheless, these figures suggest that those who experience alexithymia are more likely to experience concomitant suicide ideation.

Finally, one study assessed the longitudinal relationship between alexithymia and suicide ideation. Links et al. (2012) found that alexithymia did not significantly predict suicide ideation measured post discharge from hospital (OR=1.02, CI=0.99–1.05, *p*=.1809) nor did alexithymia predict change in suicide ideation scores between baseline and one-month follow-up (*B* = 0.12, *p*=.227). There is therefore currently no evidence that alexithymia is associated with suicide ideation over time.

#### 3.4.2 Suicide ideation and alexithymia: multivariate relationship

A total of seven studies examined the multivariate relationship between alexithymia and suicide ideation (see Table 3). The most common confounding variables included in analyses were age (*k* = 4), gender (*k* = 4) and depression severity (*k* = 4). As can be seen in Table 3 it appears that the relationship between alexithymia and suicide ideation is robust when accounting for various demographic and clinical variables. The exception to this is the study conducted by Wood et al. (2010); when depression, hopelessness and worthlessness were controlled for, the relationship between alexithymia and suicide ideation was no longer significant. This study found that only worthlessness made a unique significant contribution to the model, with patients who reported worthlessness being 91% more likely to report suicide ideation than those who did not report feelings of worthlessness.

**Table 3:** Multivariate associations between alexithymia and suicide ideation.

Study	Bivariate association	Multivariate association	Control variables
Chang et al. (2016)	–	Standardised *B* = 0.15^⁎⁎⁎^	Demographic variables, parental bonding, personality characteristics, mental disorder
De Berardis (2008)	*r* = 0.62*	*r* = 0.75^⁎⁎⁎^	Demographic variables
De Berardis (2017)	*r* = 0.80*	*r* = 0.72^⁎⁎⁎^	Demographic variables
Hamilton (2003)	*r* = 0.53^⁎⁎⁎^	R^2^ change = 0.06^⁎⁎^	Anxiety
Hintikka et al. (2004)	OR: 1.06 (95% CI = 1.05–1.08)*	OR: 1.03 (95% CI = 1.01–1.05)*	Demographic variables, psychosocial and economic risk factors, depression
Kim et al. (2016)	*r* = 0.36^⁎⁎⁎^	*r* = 0.31^⁎⁎^	Depression
Wood et al. (2010)	Unstandardized *B* = 0.35*	Unstandardized *B* = 0.02†	Depression, hopelessness, worthlessness

*p<.05.

**p<.01.

***p<.001.

† Non significant.

A meta-regression was conducted using the Knapp–Hartung variance estimator (Harbord and Higgins, 2008) to explore characteristics which might account for the high degree of inconsistency across studies. The results of this meta-regression should be interpreted with caution since they were planned post-hoc and meta-regression is prone to Type 1 error (Higgins and Thompson, 2004). Specifically, age (25 years and under, *K* = 5 vs. over 25 years, *K* = 10) and geographic location (Eastern, *K* = 4 vs. Western, *K* = 11) were explored as potential moderators. Neither age (β= 0.72, 95% CI = −0.35–0.49, *p*= .714) nor geographic location (β=0.68, 95% CI = −0.37–0.51, *p*=.743) were significant moderators in the relationship between alexithymia and suicide ideation. Moreover, neither moderator explained much of the between-study variance with I^2^ values still remaining high (age=94.4%, geographic location=93.9%).

#### 3.4.3 Suicide ideation and alexithymia subscales

Twelve studies assessed the relationship between subscales of TAS-20 and suicide ideation and a further two studies assessed the bivariate relationship between subscales of TAS-26 and suicide ideation (see Table 4).

**Table 4:** Suicide ideation and alexithymia subscales: Bivariate and multivariate relationships.

Study	Alexithymia measure	DIF bivariate relationship	DIF multivariate relationship	DDF bivariate relationship	DDF multivariate relationship	EOT bivariate relationship	EOT multivariate relationship	Control variables
Carano et al. (2012)	TAS-20	–	Standardised *B* = 0.54*	–	Standardised *B* = 0.21*	–	Non-significant – no values given†	Depression
De Berardis (2008)	TAS-20	–	pr=0.72⁎⁎⁎	–	pr=0.64⁎⁎⁎	–	pr=0.20†	Demographic variables
De Berardis (2013)	TAS-20	–	Standardised *B* = 0.31⁎⁎	–	Non-significant – no values given†	–	Non-significant – no values given†	Demographic variables, panic disorder, depression, laboratory variables, TAS-20 subscales
De Berardis (2014)	TAS-20	–	Standardised *B* = 0.54*	–	Non-significant – no values given†	–	Non-significant – no values given†	Demographic variables, OCD, depression, laboratory variables, TAS-20 subscales
De Berardis (2015)	TAS-20	–	Standardised *B* = 0.29⁎⁎⁎	–	Non-significant – no values given†	–	Non-significant – no values given†	Demographic variables, OCD, depression, responsibility attitudes, TAS-20 subscales
De Berardis (2017)	TAS-20	–	pr=0.70⁎⁎⁎	–	pr=0.53⁎⁎⁎	–	pr=0.58⁎⁎⁎	Demographic variables
Ghorbani et al. (2017)	TAS-20	*r* = 0.42⁎⁎⁎	pr=0.12†	*r* = 0.53⁎⁎⁎	pr=0.29⁎⁎⁎	*r* = 0.58⁎⁎⁎	pr=0.42⁎⁎⁎	Depression, alcohol dependence severity
Huang et al. (2016)	TAS-20	*d* = 1.03*	Standardised *B* = 0.03⁎⁎	*d* = 1.64*	–	*d* = 0.79*	–	Demographic variables, adverse life events, personality traits, suicidality factors
Izci et al. (2015)	TAS-20	*r* = 0.64⁎⁎⁎	–	*r* = 0.41⁎⁎⁎	–	*r* = 0.09†	–	–
Loas et al. (2016)	TAS-20	*r* = 0.19*	Standardised *B*= −0.23*	*r* = 0.28*	Standardised *B* = 0.23⁎⁎	*r* = 0.00†	Non-significant – no values given†	Demographic variables, depression, anhedonia, TAS-20 subscales, laboratory variables
Sakuraba et al. (2005)	TAS-26	Significant – no values given⁎⁎⁎	–	Significant – no values given⁎⁎⁎	–	Non-significant – no values given†	–	–
Sayar et al. (2003)	TAS-26	*r* = 0.44⁎⁎⁎	–	Non-significant – no values given†	–	Non-significant – no values given†	–	–
Wood et al. (2010)	TAS-20	*d* = 0.47*	–	*d* = 0.39*	–	*d* = 0.24†	–	–

EOT=Externally Oriented Thinking; DDF=Difficulty Describing Feelings; DIF=Difficulty Identifying Feelings; OCD=Obsessive Compulsive Disorder; TAS-20=Toronto Alexithymia Scale.

⁎ *p*<.05.

⁎⁎ *p*<.01.

⁎⁎⁎ *p*<.001.

† Non-significant.

A meta-analysis of the bivariate relationship between difficulty identifying feelings (DIF) and suicide ideation revealed a ‘moderate’ relationship (*r* = 0.41, 95% CI= 0.29–0.51, *k* = 6). This effect size is smaller than that found for the bivariate relationship between total alexithymia scores and suicide ideation (*r* = 0.54). Significant cross-sectional relationships between DIF and suicide ideation largely remained when demographic and clinical variables were accounted for.

A ‘moderate’ effect size was found in the meta-analysis of the relationship between difficulty describing feelings (DDF) and suicide ideation (*r* = 0.43, 95% CI = 0.24–0.59, *k* = 5). This effect size is again smaller than that found for the bivariate relationship between total alexithymia scores and suicide ideation (*r* = 0.54) but is very similar to that found for the relationship between DIF and suicide ideation (*r* = 0.41). Largely these findings remained significant when accounting for demographic and clinical variables.

The relationship between externally oriented thinking (EOT) and suicide ideation was found to have a ‘small’ effect size according to the meta-analysis (*r* = 0.28, 95% CI = 0.09–0.46, *k* = 5). This effect size is much smaller than those found between alexithymia total score (*r* = 0.54), DIF (*r* = 0.41) or DDF (*r* = 0.43) subscales and suicide ideation. Additionally, this relationship does not appear robust when accounting for clinical and demographic variables.

### 3.5 The relationship between suicide behaviour and alexithymia

#### 3.5.1 Suicide behaviour and alexithymia: bivariate relationship

**Fig. 5:**
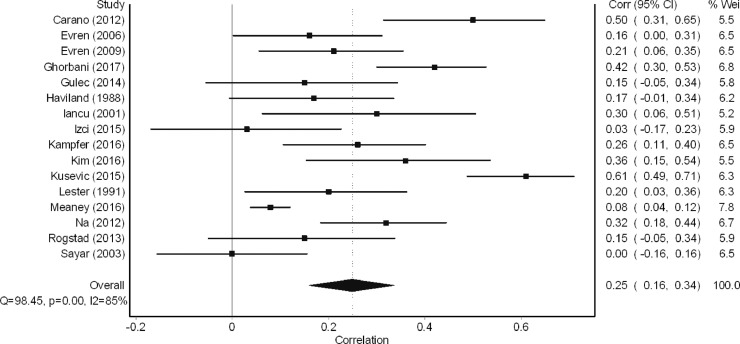
Meta-analysis of relationship between alexithymia and suicide behaviour.

A total of 17 studies examined the bivariate relationship between alexithymia and suicide behaviour. Of these, 16 studies were eligible for inclusion in the meta-analysis. The meta-analysis revealed a small effect size in the relationship between alexithymia and suicide behaviour (*r* = 0.25, 95% CI = 0.16–0.34, *k* = 16, Fig. 5). When considering study designs, it was found that effect sizes were largest for between-group case-control studies (Difference in alexithymia scores; Cohen's *D* = 0.71, 95% CI =0.48–0.93. Difference in prevalence of suicide behaviours; OR=0.17, 95% CI=0.07–0.45). Effect sizes were considerably smaller for within-group cross-sectional studies (*r* = 0.11, 95% CI=0.04–0.17).

In addition to the data included in the meta-analysis, one study (Links et al., 2012) utilised a longitudinal design to examine the relationship between alexithymia at baseline and suicide attempts occurring within six months of hospital discharge. Alexithymia was not found to be a significant univariate predictor of suicide attempts (OR=0.99, 95% CI=0.94–1.03, *p*=.552).

#### 3.5.2 Suicide behaviour and alexithymia: multivariate relationship

A total of eight studies examined the multivariate relationship between alexithymia and suicide behaviour (see Table 5). The most common confounding variables included in analyses were depression severity (*k* = 4), age (*k* = 3), gender (*k* = 3) and marital status (*k* = 3). As can be seen in Table 5 control variables appears to have a mixed impact on this relationship, suggesting that the relationship between suicide behaviours and alexithymia is not robust, and instead may be accounted for by other factors.

**Table 5:** Multivariate associations between alexithymia and suicide behaviour.

Study	Bivariate association	Multivariate association	Control variables
Chang et al. (2016)	–	Standardised *B* = 0.14⁎⁎⁎	Demographic variables, parental bonding, personality characteristics, mental disorder, suicide ideation
Gulec et al. (2014)	*r* = 0.15†	Non-significant – no values given†	Demographic variables, clinical variables, character variables, childhood maltreatment
Kim et al. (2016)	*r* = 0.36⁎⁎⁎	Standardised *B* = 0.076⁎⁎	Demographic variables, depression, OCD, perfectionism
Kusevic et al. (2015)	–	Odds ratio=2.87 (95% CI=1.18–7.0)*	Demographic variables
Levi et al. (2008)	–	Standardised *B* = 0.16*	Mental pain, depression, hopelessness, life events, schizoid, loneliness
Links et al. (2012)	–	Odds ratio=0.93 (95% CI=0.87–0.99)*	Demographic variables, suicidality variables, depression, impulsivity, problem solving, clinical variables, patient-care factors
Na et al. (2013)	*r* = 0.32*	Odds ratio=2.31 (95% CI=1.08–4.97)*	Cooperativeness

⁎ *p*<.05.

⁎⁎ *p*<.01.

⁎⁎⁎ *p*<.001.

† Non-significant.

A meta-regression was conducted to explore whether age (25 years and under, *K* = 4 vs. over 25 years, *K* = 10) and geographic location (Eastern, *K* = 10 vs. Western location, *K* = 5) were potential moderators in the relationship between alexithymia and suicide behaviour. Neither age (β= 0.12, 95% CI = −0.07–0.30, *p*= .193) nor geographic location (β=0.05, 95% CI = −0.13–0.24, *p*=.524) were significant moderators in the relationship between alexithymia and suicide ideation. Moreover, neither moderator explained much of the between-study variance with I^2^ values still remaining high (age=67.7%, geographic location=75.9%).

#### 3.5.3 Suicide behaviour and alexithymia subscales

Eight studies assessed the relationship between subscales of TAS-20 and suicide behaviour and a further one study assessed the relationship between subscales of TAS-26 and suicide behaviour (see Table 6).

**Table 6:** Suicide behaviour and alexithymia subscales: Bivariate and multivariate relationships.

Study	Alexithymia measure	DIF bivariate relationship	DIF multivariate relationship	DDF bivariate relationship	DDF multivariate relationship	EOT bivariate relationship	EOT multivariate relationship	Control variables
Evren (2006)	TAS-20	*d* = 0.49*	–	*d* = 0.49*	–	*d*= - 0.11†	–	–
Ghorbani et al. (2017)	TAS-20	–	Non-significant – no values given†	–	Non-significant – no values given†	–	Unstandardized *B* = 0.14⁎⁎⁎	Demographic variables, alcohol dependency, depression, emotion regulation, TAS-20 subscales
Gulec et al. (2014)	TAS-20	*d* = 0.37*	–	*d* = 0.37†	–	*d*= −0.33†	–	–
Izci et al. (2015)	TAS-20	*r* = 0.06†	–	*r* = 0.04†	–	*r* = 0.04†	–	–
Loftis (2014)	TAS-20	*r* = 0.28⁎⁎⁎	–	*r* = 0.14⁎⁎⁎	–	*r* = 0.07†	–	–
Meaney et al. (2016)	TAS-20	*r* = 0.10⁎⁎⁎	–	*r* = 0.08⁎⁎⁎	–	*r*= −0.02†	–	–
Na (2013)	TAS-20	*d* = 0.72*	OR=1.07 (95% CI=1.01–1.13)*	*d* = 0.72*	OR=1.20 (95% CI=1.09–1.32)⁎⁎⁎	*d* = 0.36*	–	Cooperativeness
Rogers (2015)	TAS- 20	*r* = 0.08*	–	*r* = 0.15†	–	*r* = 0.13†	–	–
Sayar et al. (2003)	TAS-26	*d* = 0.17†	–	*d* = 0.09†	–	*d*= −0.32†	–	–

EOT=Externally Oriented Thinking; DDF=Difficulty Describing Feelings; DIF=Difficulty Identifying Feelings; TAS-20=Toronto Alexithymia Scale.

^⁎⁎^*p*<.01.

⁎ *p*<.05.

⁎⁎⁎ *p*<.001.

† Non-significant.

A meta-analysis revealed a ‘small’ effect size in the relationship between DIF and suicide behaviour (*r* = 0.16, 95% CI = 0.09–0.23, *k* = 8). This effect size is much smaller than that found for the bivariate relationship between alexithymia total score and suicide behaviour (*r* = 0.30). Furthermore, this effect size is much smaller than that found for the relationship between DIF and suicide ideation (*r* = 0.41). There are limited and mixed results on the impact that clinical and demographic variables have on this relationship.

There was also a ‘small’ effect size for the relationship between DDF and suicide behaviour (*r* = 0.15, 95% CI = 0.08–0.21, *k* = 8). This effect size is very similar to that found between DIF and suicide behaviour (*r* = 0.16), but is much smaller than the relationship found between DDF and suicide ideation (*r* = 0.43). There are mixed findings regarding the impact of clinical and demographic variables on this relationship.

Finally, the meta-analysis did not support the hypothesis of a bivariate relationship between EOT and suicide behaviour (*r* = 0.00, 95% CI = −0.07–0.08, *k* = 8). This effect size is much smaller than those found for the subscales of DIF and DDF with suicide behaviour (*r* = 0.16 and *r* = 0.11) and is also much smaller than the relationship between EOT and suicide ideation (*r* = 0.28). There is not enough data to assess the impact of clinical and demographic variables on this relationship.

### 3.6 The relationship between suicide risk and alexithymia

A total of three studies examined the relationship between alexithymia and suicide ‘risk’. All three studies found evidence to suggest that alexithymia is related to suicide risk. Iancu et al. (2001) found a moderate correlation between alexithymia and the suicide risk scale (*r* = 0.3, *p*<.05). Furthermore, Hamilton (2003) found a large correlation between alexithymia and the suicide probability scale (*r* = 0.53, *p*<.001). Finally, Loftis (2014) found that alexithymia significantly predicted suicide risk as measured by the suicide behaviours questionnaire revised (β = 0.21, *p*<.001). Specifically, Loftis (2014) found that alexithymia accounted for 4% of the variance in suicide risk. Furthermore, of the participants who classified as at risk for suicide, 64.4% of these scored above the cut-off point for alexithymia. Loftis also found that suicide risk was more closely related to difficulty identifying feelings (*r* = 0.28, *p*<.001) and difficulty describing feelings (*r* = 0.14, *p*<.001) than it was with externally oriented thinking (*r*= −0.07, *p*=.12).

### 3.7 Summary

Table 7 shows a summary of the bivariate relationships between alexithymia and suicide ideation and behaviour. The review found a large effect size for the relationship between alexithymia and severity of suicide ideation (*r* = 0.54, 95% CI=0.40–0.65). Furthermore, the review found moderate effect sizes for the relationship between difficulty identifying feelings (DIF, *r* = 0.41, 95% CI=0.29–0.51) and difficulty describing feelings (DDF, *r* = 0.43, 95% CI=0.24–0.59) subscales with suicide ideation. Finally, the review found small effect sizes for the relationship between alexithymia and suicide behaviour (*r* = 0.25, 95% CI=0.16–0.34), externally oriented thinking (EOT) and suicide ideation (*r* = 0.28, 95% CI=0.09–0.46), and also between suicide behaviour and DIF (*r* = 0.16, 95% CI=0.09–0.23) and DDF (*r* = 0.15, 95% CI=0.08–0.21) subscales. The review found no evidence for a relationship between EOT and suicide behaviour (*r* = 0.00, 95% CI=−0.07–0.08).

**Table 7:** Summary of bivariate relationships with suicide ideation and suicide behaviour.

	Suicide ideation		Suicide behaviour	
	r	95% CI	r	95% CI
**Alexithymia (overall scores)**	0.54*	0.40 - 0.65	0.25*	0.16 - 0.34
**Difficulty identifying feelings**	0.41*	0.29 – 0.51	0.16*	0.09 - 0.23
**Difficulty describing feelings**	0.43*	0.24 – 0.59	0.15*	0.08 – 0.21
**Externally oriented thinking**	0.28*	0.09 – 0.46	0.00	−0.07 – 0.08

⁎ p<.05.

## 4 Discussion

### 4.1 Summary of results

The aim of this review was to (1) assess the relationship between alexithymia and its subcomponents and suicidal ideation in adults, (2) assess the relationship between alexithymia and its subcomponents and suicidal behaviour in adults and (3) compare the relationships between alexithymia and suicide ideation / behaviour.

This review found a large effect size in the meta-correlation between alexithymia and suicide ideation (0.54) and a medium effect size in the meta-correlation between alexithymia and suicide behaviour (0.25). These effect sizes echo research which has found existing risk factors for suicide ideation and behaviour such as psychotic disorders, substance use disorders and attention deficit hyperactivity disorder (Franklin et al., 2017). This therefore represents a need to better understand the nature of the relationship between alexithymia and suicide ideation and behaviour and may outline alexithymia as a new treatment target.

This review found a stronger relationship between alexithymia, and all subcomponents, with suicide ideation than with suicide behaviour. This study therefore supports the findings of May and Klonsky (2016) who assert that the risk factors for suicide ideation differ from those for suicide behaviour. This finding is in line with other research which has found that emotion dysregulation more broadly has been found to be more closely correlated with suicide ideation than suicide behaviour (Anestis et al., 2011, Zlotnick et al., 1997, 2003). Despite emotion dysregulation having a weaker relationship with suicide behaviour, there is nonetheless an association between emotion dysregulation and suicide behaviour. This has been explained by evidence that people who have difficulty regulating their emotions are only at greater risk of engaging in suicide behaviour if they have also engaged in repetitive self-injury (Law et al., 2015). This may explain the findings in the current review, especially given that several studies have found a relationship between alexithymia and non-suicidal self-injury (Norman and Borrill, 2015).

Further to this, this review found greater evidence for a relationship between suicide ideation and behaviour and the subscales of difficulty identifying feelings (DIF) and difficulty describing feelings (DDF) than with the externally oriented thinking (EOT) subscale. As discussed below, the impact of depression on the relationship between alexithymia and suicide ideation and behaviour is as yet unclear. However, in a recent meta-analysis, it was found that only the DIF and DDF components of alexithymia correlated with depression, and not the EOT component (Li et al., 2015). Assuming that depression has an impact on the relationship between alexithymia and suicide ideation and behaviour, this may help to explain the findings in this review that EOT was not as closely related to suicide ideation or behaviour as DIF and DDF subscales. Furthermore, the EOT component of the TAS-20 has frequently been found to have low internal consistency (Thorberg et al., 2010). This notion is supported by the fact that several studies have not been able to replicate the three factor structure of TAS-20 in a range of populations (Haviland, 1996, Müller et al., 2003, Zech et al., 1999).

The findings in this review regarding multivariate associations between alexithymia, subscales and suicide ideation and behaviour are somewhat unclear. This review found some evidence that demographic variables such as age, gender and location did not have any impact on the relationship between alexithymia and both suicide ideation and behaviour. Despite this, the review has highlighted inconsistencies in whether clinical variables, particularly depression, have an impact on the relationship between alexithymia and suicide ideation and behaviour or not.

To date, there has been much debate about the distinction between alexithymia and depression, with some even suggesting that subscales of alexithymia do no more than measure the pre-defined concept of depression (Cohen, 1989). This review found that the relationship between alexithymia and suicide ideation remained robust when depression was controlled for. However, when depression was controlled for in the relationship between alexithymia and suicide behaviour, the relationship did not appear as robust. Studies included in this review tended to measure a number of confounding variables, making it difficult to disentangle the specific impact of depression upon this relationship. Future research should aim to delineate the precise role of depression in the relationship between alexithymia and suicide ideation and behaviour.

### 4.2 Strengths, limitations and areas for future research

The present review involved a comprehensive search of the literature for evidence relating to the relationship between alexithymia and suicide ideation and behaviour. This review has also used meta-analyses to help synthesise the data and describe the current findings in the literature regarding the relationship between alexithymia and suicide ideation and behaviour. This review encompasses research from a variety of sources including peer-review journals and theses, which may go some way to alleviating a publication bias in the results.

Despite this, the review excluded studies that were not written in English which may limit the findings’ generalisability to non-Western cultures. However, the review does include several studies which originate from non-English speaking countries. Further, the review is limited in its exclusion of studies which examine child and adolescent samples. Future reviews could therefore focus on the relationship between alexithymia and suicide ideation and behaviour in child and adolescent populations.

Another limitation of the present review is the relatively high I^2^ levels of inconsistency within meta-analyses. This statistic is indicative of high levels of between-study variance, suggesting that differences in the design of studies may account for the differences in correlation coefficients. Despite relatively high levels of inconsistency, others have still advocated for the use of a meta-analysis, due to the approach being able to quantify and comment on levels of inconsistency (Ioannidis et al., 2008).

High levels of heterogeneity are particularly pertinent to studies examining the relationship between alexithymia and suicide behaviour, as the included measures of suicide behaviour varied considerably, with few studies utilising validated measures to assess suicide behaviour. Future research investigating the relationship between alexithymia and suicide ideation and behaviour should aim to utilise a validated measure of suicide ideation and/or behaviour, in order to ensure that measurement is accurate, reliable and valid.

In this review statistical testing of moderating effects of clinical variables were not possible due to there being small numbers in each subgroup, a limited number of studies included in the review, and due to such analysis being post-hoc in nature and therefore lending itself to a Type 1 error (Higgins and Thompson, 2004, Thompson and Higgins, 2002). Therefore important distinctions in the relationship between alexithymia and suicide ideation and behaviour may not have been discovered in the current study. Once a more substantial body of research in this area has been established, future reviews should aim to incorporate sub-group analysis into the design of the review, allowing for comparison of, for example, different clinical diagnoses.

The majority of studies included in this review included only one time point of data collection. However, in order to ascertain whether a causal relationship exists between alexithymia and suicide ideation and behaviour, temporality and directionality must be examined to decipher which phenomenon occurs first (Bradford, 1965). Future research should therefore aim to examine the prospective relationship between alexithymia and suicide ideation and behaviour. Furthermore, to establish a causal relationship between alexithymia and suicide ideation and behaviour, it is recommended that future research utilise experimental designs which aim to reduce alexithymia before assessing its impact on suicidal ideation and behaviour outcomes.

Finally, this review was inconclusive regarding which factors may facilitate or hinder the relationship between alexithymia and suicide ideation and behaviour. Future research should aim to control only for depression in the analysis of the relationship between alexithymia and suicide ideation and behaviour given the concept's strong overlap with both alexithymia and suicide ideation and behaviour. Furthermore, it is important for research to assess for self-harm as a possible mechanism through which alexithymia might lead to suicide behaviour.

### 4.3 Clinical implications

The implications of this study should be interpreted with caution due to the high levels of heterogeneity found in the review. Despite this, the present findings have several important implications for clinical practice. Firstly, this review has found that alexithymia is closely related to suicide ideation. This suggests that clinicians working with suicidal patients should attempt to screen patients for alexithymia. Alexithymia may often present with psychosomatic symptoms, so it is important for clinicians to be aware of and assess appropriately for alexithymia in suicidal patients (Taylor, 1984).

Screening for alexithymia is integral to ascertaining the best treatment option for patients. It has been frequently found that individuals who experience alexithymia may struggle to engage with talking therapies due to their lack of emotional insight (Grabe et al., 2008) which can subsequently lead to boredom for the psychotherapist, undermining the therapeutic relationship (Taylor, 1984) and also higher drop-out rates (Pierloot and Vinck, 1977). Despite this, it has been suggested that talking therapies provide a useful intervention for people with alexithymia, but only when therapy is tailored to tackle the specific needs that a person experiencing alexithymia may exhibit (Krystal, 1979). For instance, it has been suggested that people who struggle with emotional clarity may benefit from more skills-based therapies as opposed to therapies which are insight-oriented. Such behavioural therapies have been found to improve competency in identifying and describing feelings (Kennedy and Franklin, 2002).

Given that this review found a stronger association between the difficulty identifying and describing feelings components of alexithymia than the externally oriented thinking component, it is suggested that clinicians should aim to focus on these difficulties before the patient engages in talking therapy, in order to maximise effectiveness of talking therapy. There is some evidence to suggest that teaching elements of emotional intelligence may help to reduce severity of alexithymia (Amani et al., 2014). Engaging in educational material about emotional intelligence may therefore help reduce the severity of alexithymia in patients to enough of a degree to enable them to actively participate in and benefit from talking therapies.

This review has synthesised and analysed the results of 34 studies which have investigated the relationship between alexithymia and suicide ideation and behaviour. The review has found stronger evidence for a relationship between alexithymia and suicide ideation than with suicide behaviour. Further, the review found the difficulty identifying and describing feelings subcomponents of alexithymia were more closely linked with suicide ideation and behaviour than the subcomponent which relates to externally oriented thinking.
